# Automated pipeline for denoising, missing data processing, and feature extraction for signals acquired via wearable devices in multiple sclerosis and amyotrophic lateral sclerosis applications

**DOI:** 10.3389/fdgth.2024.1402943

**Published:** 2024-09-27

**Authors:** Luca Cossu, Giacomo Cappon, Andrea Facchinetti

**Affiliations:** Department of Information Engineering, University of Padova, Padova, Italy

**Keywords:** smartwatches, processing, feature extraction, health data, wearable devices, long-term health monitoring

## Abstract

**Introduction:**

The incorporation of health-related sensors in wearable devices has increased their use as essential monitoring tools for a wide range of clinical applications. However, the signals obtained from these devices often present challenges such as artifacts, spikes, high-frequency noise, and data gaps, which impede their direct exploitation. Additionally, clinically relevant features are not always readily available. This problem is particularly critical within the H2020 BRAINTEASER project, funded by the European Community, which aims at developing models for the progression of Multiple Sclerosis (MS) and Amyotrophic Lateral Sclerosis (ALS) using data from wearable devices.

**Methods:**

The objective of this study is to present the automated pipeline developed to process signals and extract features from the Garmin Vivoactive 4 smartwatch, which has been chosen as the primary wearable device in the BRAINTEASER project. The proposed pipeline includes a signal processing step, which applies retiming, gap-filling, and denoising algorithms to enhance the quality of the data. The feature extraction step, on the other hand, utilizes clinical partners' knowledge and feedback to select the most relevant variables for analysis.

**Results:**

The performance and effectiveness of the proposed automated pipeline have been evaluated through pivotal beta testing sessions, which demonstrated the ability of the pipeline to improve the data quality and extract features from the data. Further clinical validation of the extracted features will be performed in the upcoming steps of the BRAINTEASER project.

**Discussion:**

Developed in Python, this pipeline can be used by researchers for automated signal processing and feature extraction from wearable devices. It can also be easily adapted or modified to suit the specific requirements of different scenarios.

## 1 Introduction

Multiple Sclerosis (MS) and Amyotrophic Lateral Sclerosis (ALS) are chronic conditions characterized by a progressive decline in motor and cognitive neurological functions. Although they are distinct diseases, they present similar challenges for patients and the healthcare system (1). Individuals with either condition must transition between receiving care in clinical facilities and managing daily care at home to monitor disease progression and treat acute episodes. This constant physical and psychological burden, coupled with an uncertain future, is shared by both patient groups. Clinicians, on the other hand, require tools that can effectively support patients with ALS and MS by providing personalized therapeutic decisions based on the patient’s conditions, identifying critical interventions, and providing insight into the status of the disease and the overall clinical situation. In recent years, significant efforts have been made to estimate the progression of ALS and MS and to develop tools that can assist both patients and clinicians in managing the disease (2, 3).

BRAINTEASER (BRinging Artificial INTelligencE home for a better cAre of amyotrophic lateral sclerosis and multiple SclERosis) (www.brainteaser.health) is a project funded by the European Horizon 2020 initiative, which aims to deploy Artificial Intelligence (AI)-based technologies for the daily home care of MS and ALS. In this context, AI is considered a key element in meeting the needs of both patients and clinicians. Specifically, AI methodologies can be utilized to analyze the progression of MS and ALS, allowing for the capture and handling of patients’ inter-variability, and providing tools for forecasting disease evolution (4). For AI methods to be effective, they need to be trained on large quantities of heterogeneous data from various sources, such as patient-specific medical history, environmental data, and signals potentially derived from commercially available wearable devices. Currently, wearable devices are widely available and they are becoming an essential instrument to monitor patients’ health-related signals in an almost continuous, noninvasive, and painless way, moving the collection process from limited controlled in-clinic sessions to daily life. Among wearable devices, commercial smartwatches allow users to easily track several important signals such as heart rate, step counts, physical exercise, and pulse oximetry, which can be used to evaluate the general health condition of the wearer (5). These functionalities can be particularly beneficial for patients with chronic diseases, who can use them to monitor the status of their disease, while clinicians can utilize these data to gain insight into disease progression. However, there are two main problems with using commercially available smartwatches to collect health-related signals. The first is that these health signals often cannot be directly used as provided by the device, mainly because of the noise of collected signals and elements tied to the use of the device itself. In fact, the wearer’s movement during usage might lead to artifacts in the signals, in the form of spikes, high-frequency noise, or gaps. Moreover, the device must be user-friendly and with an appropriate form factor, to avoid dropouts in use, thus ensuring continuous data collection. Finally, the device battery has to be recharged periodically, inevitably leading to data loss in that specific period. The second problem is that clinically relevant features, essential for monitoring chronic diseases such as ALS and MS, are not currently available through these devices. To solve these problems, many solutions have been proposed in the literature to process and analyze wearable data, but most of them have been applied only to non-consumer/experimental devices or to signals with different characteristics than the one collectible from consumer wearable devices like smartwatches (6, 7). Some previous works have focused on the processing of wearable data. For example, the works by Beyer et al. (8) and Vega et al. (9) present two processing pipelines for various wearabale devices, but lack the support for the Garmin Vivoactive 4 smartwatch, used for the project. Others like the works by Bent et al. (10) do not support all the required signals or don’t extract the features needed for the study. Lastly, the work by Foell et al. (11), while supporting the specific device, requires the data to be in the raw extracted format, which was not available during the project. Therefore, this work aims to present the automated pipeline to process health-related signals and extract clinically relevant features for ALS and MS that we developed within the BRAINTEASER project, in which signals are acquired via Garmin Vivoactive 4 smartwatch. Our solution has been meticulously crafted to meet the distinct needs, peculiarities, and requirements of the BRAINTEASER project. Briefly, the automated pipeline is composed of two steps: the signal processing step applies retiming, gap filling and denoising algorithms to improve data quality, whereas the feature extraction step selects the most important variables for ALS and MS according to clinical partners’ knowledge and feedback. The proposed automated pipeline, developed in Python, can be of help to researchers for the preliminary automated processing of the large amount of data that can be collected from wearable devices and can be easily adapted/modified to suit the specific needs of each scenario.

The structure of the paper is the following. We will start by presenting the BRAINTEASER project and its aims, and the rationale behind the selection of the Garmin Vivoactive 4 smartwatch and, by exploiting the feedback received from the clinical teams in the project, we will show the procedure we applied to identify the most useful signals and features. Then, we will illustrate in detail the automated pipeline we developed to obtain signals with improved quality and extract relevant features for AI-based models employed in BRAINTEASER. Finally, we will conclude by thoroughly defining all the extracted features, and in the last section, we will present the effectiveness of the automated pipeline by evaluating the result of its application to pivotal beta testing sessions.

## 2 Materials and methods

### 2.1 Wearable device selection for ALS/MS model development in BRAINTEASER

The BRAINTEASER project aims at exploiting clinically relevant features extracted from wearable devices to feed AI-based models to monitor and predict the progression of ALS and MS chronic diseases. Before selecting the wearable device, there was the need to better understand which signals could be potentially relevant, from the clinical point of view, for the two considered diseases.

#### 2.1.1 Wearable device data of interest for ALS/MS AI-based model development

Both ALS and MS have multiple degradation effects on many vital functions of patients, especially related to respiration, blood oxygenation, and fatigue. These symptoms have been studied and analyzed in recent years by many research teams, to better understand their connection with the disease and its progression. Heart rate and related features have been identified as significantly different between individuals with each respective disease and healthy subjects (12–14). Thus this signal has been identified as relevant to track in both diseases, to investigate possible insight into patients’ status, even in the early stages. Respiration rate has proven to be an easy-to-collect signal that is highly correlated to more invasive clinical tests to predict survival in ALS patients (15, 16), and for MS respiratory dysfunction is one of the main outcomes of the disease (17). Moreover, sleep time is critical for both diseases. In fact, it has been shown how sleep quality and fatigue are relevant for both diseases, and $\mathrm{Sp}O_{2}$ tracking during sleep can show important patterns and apnoea periods (18–20). The final clinical relevance of HRV and SpO_2_ features as key info to monitor the progression of ALS and MS diseases will be available only at the end of the AI-based model development stage. However, it is important to note that the ultimate clinical relevance of HRV and SpO_2_ features as crucial information for monitoring the progression of ALS and MS diseases will only be determined at the conclusion of the development stage of the AI-based models.

To further validate these findings with hands-on input, we asked the clinical partners of the BRAINTEASER project to provide a list of desired features to collect from the wearable device, their priority, and the ideal sampling rate. For MS, Fondazione Istituto Neurologico Nazionale Casimiro Mondino (IT) and Servicio Madrileño de Salud (ES) are the clinical partners involved in BRAINTEASER and their teams identified a very high priority for activity-related signals, such as daily steps, burnt calories, and the raw accelerometer data. They also identified the tracking of respiration rate and $\mathrm{Sp}O_{2}$ during sleep as crucial to monitor the patient’s disease status. For ALS, the clinical partners (University of Turin (IT), Instituto De Medicina Molecular João Lobo Antunes (PT), and Servicio Madrileño de Salud (ES)) identified respiration rate and $\mathrm{Sp}O_{2}$ as crucial too, with the addition of the heart rate and beat-to-beat intervals with the request of the highest sampling rate possible. This list of desired signals is in line with the literature and allowed us to define a list of candidate wearable devices compliant with both clinical and technical requirements.

#### 2.1.2 Candidate wearable devices and critical requirements for their integration

The clinical input provided expert-based information for identifying the device’s requirements. Subsequently, a more technical analysis of the available devices was conducted. We focused on wrist-worn devices because of their ease of use, small impact on the patient’s life, wide availability, and ability to collect signals such as heart rate, sleep patterns, and blood oxygen concentration. We conducted a comprehensive literature search in order to identify the devices utilized in similar studies. Additionally, we thoroughly explored the manufacturer websites to obtain a more profound understanding of the capabilities and functionalities of each device.

Table 1 reports the devices that have been considered for the project together with the pros and cons of their potential adaptation. The considered devices collect all the required signals. As such, the device of choice has been selected revolving around the maximization of the battery life, user experience, and possibility of accessing the collected data via a dedicated Application Programming Interface (API) or Software Developer Kit (SDK). Indeed, while a short battery life would imply a high burden for the patient to charge the device and data loss during the charging period, poor user experience threatens user compliance to wear the device and the absence of an API/SDK undermines the possibility of automatically collecting data and eases the development of AI-based methodologies within the project.

**Table 1:** Compared wearable devices with a brief summary of the identified advantages and disadvantages.

Brand & Model	Advantages	Disadvantages
Garmin Vivoactive 4/4s	Extensive health, sleep & stress tracking sensors, including pulse-oximeter, long battery life (8 days without GPS)	Inbuilt GPS not useful for the project requirements
ASUS VivoWatch BP	Extensive health, sleep & stress tracking, medical grade ECG and PPG sensors, excelling battery life, blood pressure measurement	Small screen, the device’s size is bulky due to ECG sensor spot for the finger at the front, which is not required for the project
Fitbit Versa 2	Extensive health, sleep & stress tracking sensors, including pulse-oximeter, good battery life	Inbuilt GPS not useful for the project requirements. No direct Bluetooth SDK available, only web API
Withings ScanWatch	Extensive health, sleep & stress tracking sensors, including pulse-oximeter and ECG sensor, very long battery life (up to 30 days)	Hybrid analog/smartwatch with a small digital display
Polar Ignite	Health, sleep, fitness, activity and & very accurate HR tracking sensors, light weight, slender design	Relatively short battery life (4 days), no pulse-oximeter

After considering all previously listed aspects, the BRAINTEASER project agreed to select the Garmin Vivoactive 4/4s as the one that best fits the clinical and technical needs.

#### 2.1.3 Garmin Vivoactive 4/4s

The Garmin Vivoactive 4/4s (Garmin Ltd, Olathe, KS, USA) is a smartwatch that comes in two sizes and allows collecting all the signals identified in Section 2.1.1. It guarantees up to 8 days of battery life, which lowers the burden on the patient. The availability of two form factors improves the range of patients who will be able to wear the device without aesthetic and bulkiness concerns. Data collection can be automated via either via web API or via dedicated SDK, which would allow direct Bluetooth Low Energy (BLE) communication with the device from a mobile application. It is important to note that the device is not intended for clinical use, and the accuracy of the measurements was not directly quantified in this study. Our focus has been on exploring the potential use of a non-clinical-grade smartwatch as a means to gather relevant information for the BRAINTEASER project. Moreover, it has some important features to improve the user experience such as built-in notifications to encourage wearers healthy habits, like hourly stand time and drinking water.

Table 2 reports the sampling rate of each signal that can be collected from the Garmin Vivoactive 4/4s. Given this information on types of signals and their sampling rate, the next step was to investigate whether to use or discard their collection based on the specific needs of the BRAINTEASER project.

**Table 2:** List of health-related signals available from the Garmin Vivoactive 4/4s.

Signal	Sampling rate	Signal	Sampling rate
Steps	60 s	Accelerometer	1/4 s
Calories	60 s	Stress	10 s
Intensity minutes	60 s	$\mathrm{Sp}O_{2}$	30 s
Floors climbed	60 s	Respiration rate	10 s
Heart rate	15 s	Body battery	10 min
Beat-to-beat interval	15 s	Battery percentage	60 min

Of course, as discussed in Section 2.1.1, we retained heart rate, respiration rate, sleep, and $\mathrm{Sp}O_{2}$ given their key role evidenced by clinical partners.

As far as the accelerometer signal is concerned, its high sampling rate would potentially lead to a high amount of data management and processing. Furthermore, its continuous collection greatly reduces the battery life to a few hours. Due to these considerations, we chose to drop this signal. However, the inclusion of accelerometer data in future studies remains relevant to these diseases. Despite the difficulties associated with its ongoing collection, it has the potential to enhance the understanding of these conditions and should be considered whenever feasible. For this project, we have chosen to use steps and calories as a proxy for physical activity information since they are readily obtainable from the device. These two specific data types represent the cumulative sum of steps performed and calories burned up to the current moment.

Regarding Stress and Body battery signals, all clinical partners agreed that they would be beneficial to track. However, these signals are computed by Garmin and there is neither information nor validation on how these variables are calculated by the manufacturer, as well as no info on their clinical validity. The previous considerations led all the partners to agree on discarding these signals as well.

Finally, an additional note on the $\mathrm{Sp}O_{2}$ signal is needed. Enabling its collection for the whole day allows collecting only a few more points due to the user’s movement since the arm should be at rest, but has the drawback of critically reducing the duration of the battery life. For this reason, we chose to collect the $\mathrm{Sp}O_{2}$ signal during nighttime only.

The final list of signals to be collected, analyzed, and processed with the pipeline presented in this work (see Section 2.2 is reported in Table 3. Table 3 also reports the minimum sampling rate for each signal that is clinically relevant, according to clinical teams’ feedback (this may be higher than the minimum value provided by the device as reported in Table 2).

**Table 3:** Final list of health-related signals and the selected sampling rate exploited in the BRAINTEASER project after the device capabilities analysis and usability considerations.

Signal	Sampling rate	Notes
Steps	15 min	
Calories	15 min	
Intensity minutes	Daily summary	
Heart rate	15 s	
Beat-to-beat interval	15 s	
$\mathrm{Sp}O_{2}$	60 s	During sleep period
Respiration rate	60 s	During sleep period

### 2.2 Pipeline for signal processing and feature extraction

**Figure 1:**
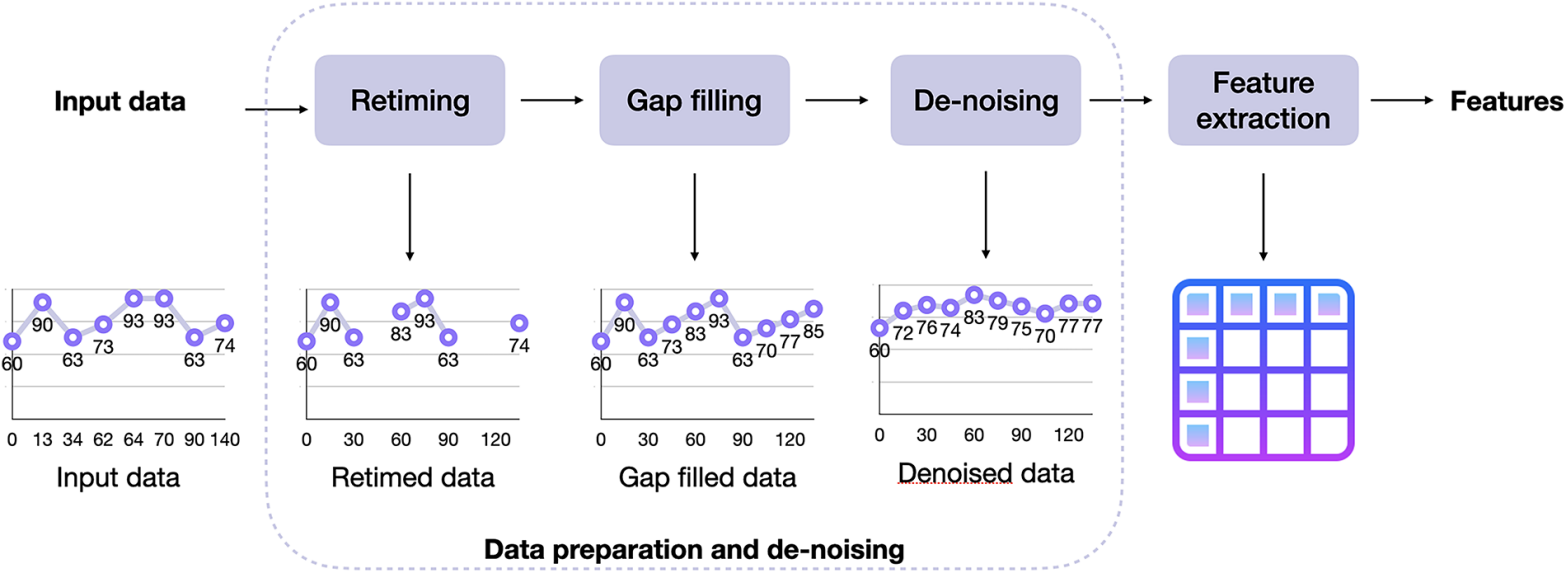
Complete processing pipeline. It can be divided into two parts: the first three blocks are devoted to signal processing, to improve the quality of the raw data by retiming the input to a fixed sampling grid, filling data gaps, and removing noise, whereas the last block performs the extraction of clinically-relevant features.

The AI models require as input clinically relevant features that are computed from the signals collected with the Garmin Vivoactive 4/4s and allow for a summary of the most important signal characteristics. In this section, we present the processing pipeline that has been designed and developed in Python aimed at extracting clinically relevant features. Figure 1 shows the overall structure of the pipeline, which is composed of three blocks of signal processing (retiming, gap filling, and denoising) aimed at making data usable to extract the features (last block).

#### 2.2.1 Wearable device data preparation and denoising

All the collected signals need to be processed before being usable for feature extraction. In our pipeline, the signal processing consists of three steps specifically tuned to deal with the characteristics of each signal.

The first step is the retiming of the signal, which aims at bringing all data points to a uniform time grid. Indeed, input data might have a non-constant sampling grid and this may bias the calculation of the features. Being our final aim of feature extraction, this aspect represents a clear problem. Retiming is implemented by an algorithm that averages all input data points to the closest output grid point. In this way, the original data are preserved and only translated to the constant time grid. The average is reduced to a sum in the case of cumulative data types, such as daily calories and steps.

The second step is gap-filling. Indeed, the data can have missing values, and the presence of missing values can bias the calculation of the features and any further analysis. Several strategies have been investigated to reliably fill data gaps. Since a priori information on wearable-derived data was not available, it has been decided to employ a simple but effective strategy, i.e., fill missing values by linear interpolation of nearby values. This choice is also functional since it allows performing all the feature extraction algorithms without introducing major changes in the dynamics of the wearable-derived data. It is also worth noting that there is no difference between gaps caused by the sensor itself and by non-wearing periods. The ad-hoc procedure we developed consists first of automatically identifying fillable gaps for each input signal, then filling them by linear interpolation only those whose duration is equal or inferior to the limits reported in Table 4. These thresholds have been meticulously established through multiple iterations of a trial-and-error process aimed at striking an optimal balance between preserving signal dynamics and mitigating the introduction of excessive artificially generated values. Importantly, these values have undergone scrutiny and received approval from our clinical project partners. For instance, the respiration rate during nighttime, which is not expected to significantly change in less than 5 min, dictates a maximum time gap for filling set at 300 s. Similarly, the heart rate and beat-to-beat signal, projected to undergo negligible alterations in less than one minute during nighttime, have prompted the establishment of a maximum time gap for filling at 60 s. The blood oxygen concentration, not anticipated to experience significant shifts in less than 2 min during nighttime, has influenced the setting of a maximum time gap for filling at 120 s. Importantly, this duration deliberately maintains a lower threshold than the respiration rate, guided by valuable insights from clinical teams emphasizing the criticality of $\mathrm{Sp}O_{2}$ levels, particularly during nighttime and periods of diminished oxygen concentration.

**Table 4:** For each signal, the maximum time gap that allows linear interpolation is reported.

Signal	Maximum time gap
Steps	No gap filling
Calories	No gap filling
Heart rate	60 s
Beat-to-Beat interval	60 s
Pulse Oximetry	120 s
Respiration rate	300 s

Note that not all signals can be processed with the gap-filling procedure, e.g., steps and calorie signals skip the gap-filling block because of their non-continuous nature, both being aggregated samples over their sampling period.

Finally, the third processing step is denoising. Due to the sensor’s characteristics, some noise overlapped with the true signal is always expected. In the case of wearable devices, this might be caused, for example, by device movement on the user’s wrist, sweating, jumps, etc., which all lead to outliers and general noise in the collected data. To improve the quality of the signals before performing the feature extraction, since no a priori information on wearable-derived data was available, we decided to adopt a very robust methodology for denoising that does not require any a priori information on the wearable-derived data, i.e., a moving average algorithm. The algorithm works by calculating the average of data points within a sliding window. This sliding window moves through the time series data, and at each position, it computes the average value of the data points within the window. This process effectively smooths out variations and reduces high-frequency noise in the data, making it a suitable choice for enhancing data quality without requiring prior information about the characteristics of the wearable-derived data. The default window is three samples wide, but it can be easily customized in the code and passed as parameter to the processing functions.

#### 2.2.2 Feature extraction

The output of the data processing pipeline (i.e., the first three blocks in Figure 1) is a signal that is now suitable to be used for the extraction of clinically relevant features. Each signal has been treated separately, to exploit all the relevant characteristics that are of interest for ALS and MS. All the features have been validated by the clinical partners of the project. Similar works in the literature on the two diseases (21–23) have served as additional confirmation of the features selected in this study. All the features are computed for the full daily data.

The explored features focus on the time, frequency, and geometric domains. In particular, the latter domain is explored by resorting to the Poincaré plot, which allows easy visualization and analysis of recursive signals like heart rate. This method and the geometric features that can be extracted from the plot are widely used in heart rate variability analysis, thus those features have been included in the list of important features for the project (24, 25). In our case, the Poincaré plot has been applied to heart and respiration rate signals only.

##### 2.2.2.1 Heart rate and beat-to-beat interval features

Heart rate variability is correlated with the presence of ALS and MS (12–14), especially its frequency domain features. To extract features for this signal that can be relevant to the project, we leveraged two main packages, Neurokit (26) and HRVAnalysis (27). The packages focused on signals with higher sampling rates, and thus some adjustments before being applied were needed, such as refactoring functions and changing their parameters. For instance, some functions in the packages required a large number of points to work on, so we had to remove that constraint. Table 5 shows the list of the extracted features from the heart rate and beat-to-beat interval signals, respectively, and their description.

**Table 5:** Complete list of features for heart rate and beat-to-beat signals. The latter is derived by the inversion of the heart rate signal.

Feature	Description
HR_Baseline	The baseline heart rate.
HR_Max	The maximum heart rate.
HR_Min	The minimum heart rate.
HR_Mean	The mean heart rate.
HR_SD	The standard deviation of the heart rate.
HR_Max_Time	The time at which maximum heart rate occurs.
HR_Min_Time	The time at which minimum heart rate occurs.
HR_Trend_Linear	The parameter corresponding to the linear trend of heart rate.
HR_Trend_Quadratic	The parameter corresponding to the curvature of heart rate.
HR_Trend_R2	The quality of the quadratic model.
MeanNN	The mean of the RR intervals.
SDNN	The standard deviation of the RR intervals.
SDANN1-2-5	The standard deviation of average RR intervals extracted from n-minute segments of time series data (1, 2 and 5 by default).
SDNNI1-2-5	The mean of the standard deviations of RR intervals extracted from n-minute segments of time series data (1, 2 and 5 by default).
RMSSD	The square root of the mean of the sum of successive differences between adjacent RR intervals.
SDSD	The standard deviation of the successive differences between RR intervals.
CVNN	The standard deviation of the RR intervals divided by the mean of the RR intervals.
CVSD	The root mean square of the sum of successive differences divided by the mean of the RR intervals.
MedianNN	The median of the absolute values of the successive differences between RR intervals.
MadNN	The median absolute deviation of the RR intervals.
HCVNN	The median absolute deviation of the RR intervals (MadNN) divided by the median of the absolute differences of their successive differences (MedianNN).
IQRNN	The interquartile range (IQR) of the RR intervals.
pNN50	The proportion of RR intervals greater than 50ms
pNN20	The proportion of RR intervals greater than 20ms
HTI	The HRV triangular index: number of RR intervals divided the height of the RR intervals histogram.
SD1	Spread of RR intervals on the Poincaré plot perpendicular to the line of identity (28).
SD2	Measure of the spread of RR intervals on the Poincaré plot along the line of identity. It is an index of long-term RR interval fluctuations.
SD1SD2	The ratio between short- and long-term fluctuations of the RR intervals (SD1/SD2).
CSI	The Cardiac Sympathetic Index (29)
CVI	The Cardiac Vagal Index (29)
CSI_Modified	The modified CSI (30)
PIP	Percentage of inflection points of the RR intervals series.
IALS	Inverse of the average length of the acceleration/deceleration segments.
PSS	Percentage of short segments
PAS	Percentage of NN intervals in alternation segments
GI	Guzik’s Index
SI	Slope Index
AI	Area Index: cumulative area of sectors corresponding to points above LI divided by the total area of sectors corresponding to all points in the Poincaré plot not on LI.
PI	Porta’s Index: number of points below LI divided the number of points in Poincaré plot not on LI.
SD1d and SD1a	Short-term variance of decelerations and accelerations, respectively (31).
C2d and C2a	The contributions of heart rate decelerations and acceleration in long-term HRV, respectively (31).
SDNNd and SDNNa	Total variance of decelerations and accelerations, respectively (31).
Cd and Ca	The total contributions of heart rate decelerations and accelerations to HRV.

##### 2.2.2.2 Pulse oximeter and respiration rate features

Sleeping hours are a vital time to watch. Numerous physiological indicators, including respiratory rate and $\mathrm{Sp}O_{2}$ (32, 33), are important to monitor during sleeping. Given that blood oxygen levels and sleep quality both reflect weariness and sleep apnoea, these signals are extremely important for both disorders being studied (18–20). Furthermore, since breathing is one of the vital processes that is most commonly hampered by disease development (16, 17, 34), it is essential to monitor respiration rate and related features to identify trends and predict disease-related events in the future. For the purpose of extracting features from pulse oximeter signals, the Neurokit2 package has been used as a starting point. The extracted features from the spO2 signal concentrate on the temporal domain, with a particular emphasis on the mean characteristics of desaturation occurrences, defined as values less than 90%. The features of the respiration signal use both the time and frequency domains, with an emphasis on the timing and characteristics of breath-to-breath intervals. The full description of the features that were extracted from $\mathrm{Sp}O_{2}$ and respiration rate is reported in Tables 6 and 7, respectively.

**Table 6:** Complete list of features for pulse oximetry signal.

Feature	Description
AV	Average of the pulse oximetry.
MED	Median of the pulse oximetry.
Min	Minimum value of the pulse oximetry.
SD	Standard deviation of the pulse oximetry.
RG	SpO_2_ range (difference between the max and min value).
P	Percentile (90th)
Mx	Percentage of the pulse oximetry 90% below median oxygen saturation.
ZC	Number of zero-crossing points.
DI	Delta Index.
CA	Integral SpO_2_ below the 90% SpO_2_ level normalized by the total recording time
CT	Percentage of the time spent below the 90% oxygen saturation level.
POD	Percentage of oxygen desaturation events
${\text{AOD}}_{\max}$	The area under the oxygen desaturation event curve, using the maximum SpO_2_ value as baseline and normalized by the total recording time
${\text{AOD}}_{100}$	Cumulative area of desaturations under the 100% SpO_2_ level as baseline and normalized by the total recording time
ODI	The average number of desaturation events per hour (int).

**Table 7:** Complete list of features for respiration rate signal.

Features	Description
SDBB	The standard deviation of the breath-to-breath intervals.
RMSSD	The root mean square of successive differences of the breath-to-breath intervals.
SDSD	The standard deviation of the successive differences between adjacent
VLF	Spectral power density pertaining to very low frequency band i.e., 0 to .04 Hz by default.
LF	Spectral power density pertaining to low frequency band i.e., .04 to .15 Hz by default.
HF	Spectral power density pertaining to high frequency band i.e., .15 to .4 Hz by default.
LFHF	The ratio of low frequency power to high frequency power.
LFn	The normalized low frequency, obtained by dividing the low frequency power by the total power.
HFn	The normalized high frequency, obtained by dividing the low frequency power by total power.
SD1	A measure of the spread of breath-to-breath intervals on the Poincaré plot perpendicular to the line of identity. It is an index of short-term variability.
SD2	SD2 is a measure of the spread of breath-to-breath intervals on the Poincaré plot along the line of identity. It is an index of long-term variability.
SD2SD1	The ratio between short- and long-term fluctuations of the breath-to-breath intervals (SD2 divided by SD1).
ApEn	The approximate entropy
SampEn	The sample entropy
DFA_alpha1	The “short-term” fluctuation value generated from Detrended Fluctuation Analysis (DFA) i.e., the root mean square deviation from the fitted trend of the breath-to-breath intervals.
DFA_alpha2	The long-term fluctuation value.
alpha1_ExpRange	Multifractal DFA of short-term fluctuations. ExpRange is the range of singularity exponents, corresponding to the width of the singularity spectrum.
alpha2_ExpRange	Multifractal DFA of long-term fluctuations. ExpRange is the range of singularity exponents, corresponding to the width of the singularity spectrum.
alpha1_ExpMean	Multifractal DFA of short-term fluctuations. ExpMean is the mean of singularity exponents.
alpha2_ExpMean	Multifractal DFA of long-term fluctuations. ExpMean is the mean of singularity exponents.
alpha1_DimRange	Multifractal DFA. DimRange is the range of singularity dimensions, corresponding to the height of the singularity spectrum.
alpha2_DimRange	Multifractal DFA. DimRange is the range of singularity dimensions, corresponding to the height of the singularity spectrum.
alpha1_DimMean	Multifractal DFA. Dimmean is the mean of singularity dimensions.
alpha2_DimMean	Multifractal DFA. Dimmean is the mean of singularity dimensions.

## 3 Results

The pipeline for signal processing and feature extraction has been validated using sample data collected during the project’s development period and the initial phase of patient enrollment. This data was obtained from 10 subjects participating in the BRAINTEASER clinical trial, including both ALS and MS patients. The subjects wore the Garmin Vivoactive 4s device in an outpatient setting without any specific tasks for data collection. The subjects gave their informed consent for inclusion before they participated in the study. The study was conducted following the Declaration of Helsinki, and the protocol was approved by the Ethics Committee of A.O.U. Città della salute e della scienza di Torino (314/2021). In this work, we present the results on a representative subject with ALS, who used the device for 7 days to acquire a comprehensive data set that encompasses both daytime and nighttime periods. During this period, this individual engaged in various physical activities, which enabled the verification of the collected data’s quality and the assessment of the efficiency of the processing pipeline in eliminating potential artifacts resulting from the device’s movement on the wrist. The data presented here, as well as additional sample data, can be found in the repository linked as Supplementary Material. This data is representative of future datasets that will be collected using the wearable device, during the BRAINTEASER clinical trial and the pipeline will be further tested on the complete dataset when available.

**Figure 2:**
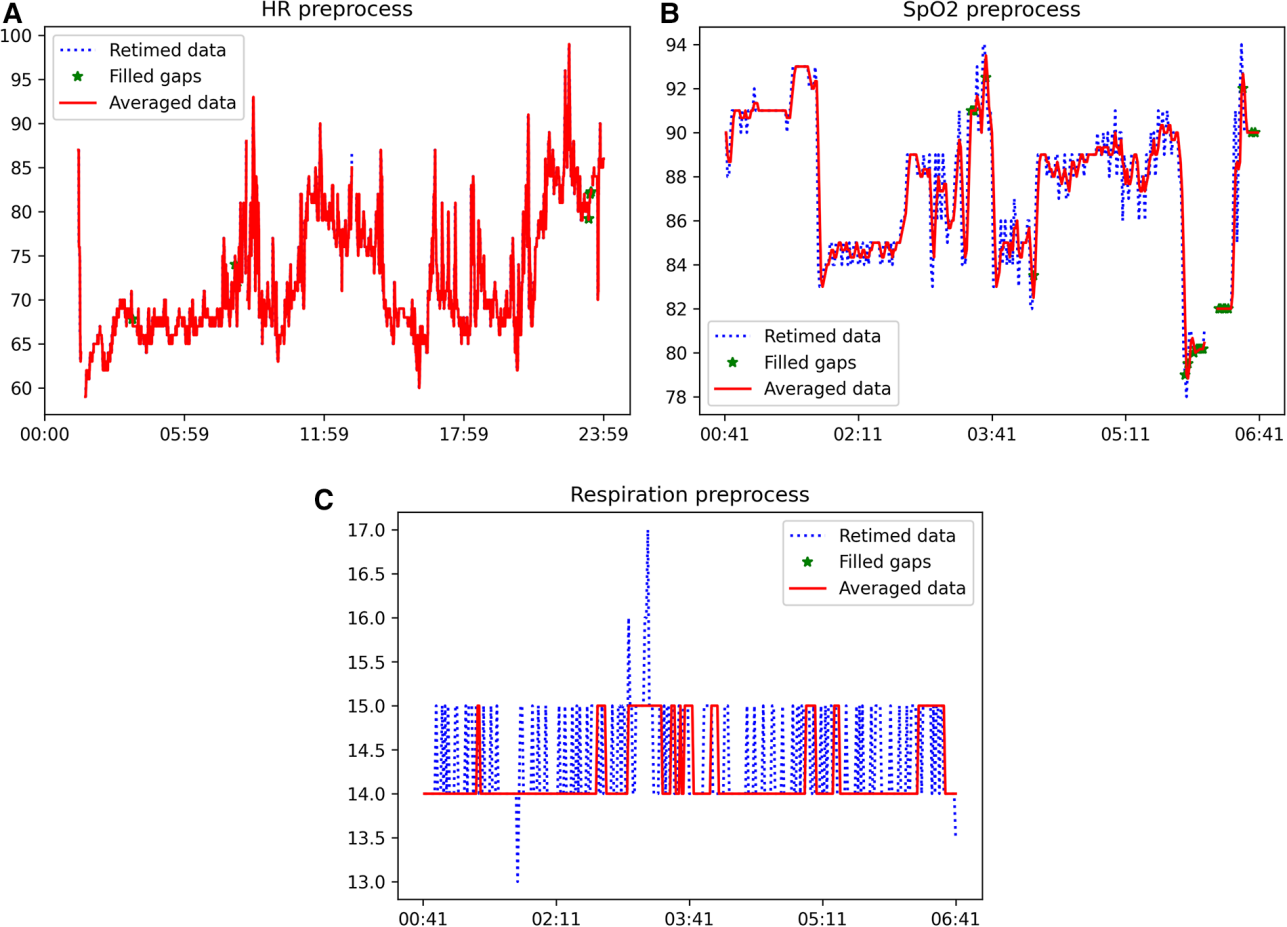
Example of the outcome of the application of the signal processing pipeline (first three blocks of Figure 1) to heart rate (top), $\mathrm{Sp}O_{2}$ (middle), and respiration (bottom) signals. The blue dashed line is the input retimed signal, the result of the gap filling is shown as green dots and the denoised signal is in red. **(A)** Example of heart rate signal after each step of the processing pipeline, **(B)** Example of $\mathrm{Sp}O_{2}$ signal after each step of the processing pipeline, **(C)** Example of respiration rate signal after each step of the processing pipeline.

The sample dataset contains some of the expected artifacts that the processing pipeline is asked to remove/correct. For instance, the sleeping time signal presents some peaks that could be a consequence of the wrong positioning of the device or compression of the sensor due to movement during sleep. Moreover, all signals present data gaps of different durations, which need to be identified and filled by linear interpolation if the gap duration is lower or equal to the limits previously. The results of the signal processing pipeline, comprising the initial three blocks outlined in Figure 1, are illustrated in Figure 2 for heart rate (top), $\mathrm{Sp}O_{2}$ (middle), and respiration (bottom) signals. Each figure depicts a segment of the available data to demonstrate the effects of each processing step. The input retimed signal is represented by the blue dashed line, the outcome of gap filling is displayed as green dots, and the denoised signal is depicted in red.

These results illustrate the varying susceptibility of different signals to artifacts. Specifically, the heart rate signal obtained from the wearable device was found to be suitable for feature extraction without the need for further processing. Conversely, the $\mathrm{Sp}O_{2}$ and respiration rate signals exhibited a significant number of missing data points and artifacts, requiring pre-processing prior to feature extraction. The proposed automatic processing pipeline demonstrates the capability to effectively identify and correct the prevalent issues in health-related signals obtained from wearable devices. The results presented in Figure 2 illustrate that the application of the signal processing steps leads to an enhancement in the usability of the acquired time-series, and subsequently, the quality of the extracted features. This is achieved through the reconstruction of missing data and the removal of artifacts through denoising.

The cleaned sample was utilized to extract sample features that will serve as input to the AI models. As an example, Table 8 presents a list of sample features that were extracted from the heart rate signal.

**Table 8:** List of the extracted features from the heart rate and beat-to-beat sample signal.

Feature	Sample value	Feature	Sample value	Feature	Sample value
Mean	72.000	PNN20	2.8220	SI	50.018
Std	6.7750	Sdsd	7.5264	AI	50.021
MaxTime	81,045	Rmssd	7.5264	PI	50.268
MinTime	6,315	CvNN	0.08856	Cvsd	0.00900
LinearTrend	0.00015	C1d	0.5691	C1a	0.4309
QuadraticTrend	0.00000	TiNN	Null	SD1d	4.0151
R2	0.28265	HTI	10.2046	SD1a	3.4939
MeanNN	836.31703	Sd1	5.3224	C2d	0.49549
SdNN	74.05639	Sd2	104.6061	C2a	0.50451
SdaNN1	73.63064	Sd1sd2	19.6538	SD2d	73.6146
SdNNI1	5.65448	Cvi	3.9498	SD2a	74.28125
SdaNN2	73.39248	Csi	19.6538	Cd	0.49568
SdNNI2	5.77449	CsiModified	8223.61677	Ca	0.50432
SdaNN5	75.46535	Lf	169.14553	SDNNd	52.13075
SdNNI5	5.52205	Hf	13.24997	SDNNa	52.58284
MedianNN	0.00000	Lf_hf_ratio	12.76573	PIP	0.32728
MadNN	47.84689	Lfnu	92.73558	IALS	0.33282
HcvNN	Infinity	Hfnu	7.26442	PSS	0.00691
IqrNN	122.99020	Total_power	1037.26770	PAS	0.00090
PNN50	0.05682	Vlf	854.87221	Baseline	61.000
GI	50.01960	Minimum	56.000	Maximum	99.000

Tables 9 and 10 show an example of the features extracted from the sleeping time signals. These focus on overall summary values and, especially for the $\mathrm{Sp}O_{2}$ signal, some of the features highlight important events such as time in desaturation and its characteristics.

**Table 9:** List of the extracted features from blood oxygen saturation signal.

Feature	Sample value	Feature	Sample value	Feature	Sample value
AV	87.613	DI	1.8754	M	34.072
MED	88.333	CA	1.2772	ODI	43.636
Min	78.833	CT	78.4848	ZC	26.000
SD	3.1469	POD	0.45758	AOD100	6.8152
RG	14.6667	AODMAX	2.7152	P	80.0278

**Table 10:** List of the extracted features from the respiration rate sample signal.

Feature	Sample value	Feature	Sample value
Mean	14.208	DFA_ALPHA1	1.2824
Var	0.16493	DFA_ALPHA1_EXPRANGE	0.73360
SDBB	0.40612	DFA_ALPHA1_EXPMEAN	0.66226
SDSD	0.06744	DFA_ALPHA1_DIMRANGE	0.68977
RMSSD	0.06744	DFA_ALPHA1_DIMMEAN	0.33348
SD1	0.04775	DFA_ALPHA2	0.83493
SD2	0.15723	DFA_ALPHA2_EXPRANGE	14.89034
SD1SD2	3.29271	DFA_ALPHA2_EXPMEAN	8.06556
AP_En	0.20313	DFA_ALPHA2_DIMRANGE	5.53735
Sam_PEn	0.08336	DFA_ALPHA2_DIMMEAN	1.59306

In this study, the validity and impact of the extracted features on model performance have not been directly evaluated. This evaluation is important to understand the effect of the pipeline on model performance. However, such an analysis would necessitate the complete development of dedicated ALS/MS models to fully leverage the features extracted by our pipeline, which is a matter of ongoing research and is out of the scope of the present manuscript. Of course, once these models are finalized within the framework of the BRAINTEASER project, future investigations will explore the impact of our processing and feature extraction steps on their performance.

## 4 Discussion

Health signals obtained from wearable devices present a valuable source of monitoring data for chronic diseases, and AI models can leverage this information to predict disease progression. In the context of the BRAINTEASER project, the Garmin Vivoactive 4 was selected as the device for training the AI models for monitoring the progression of MS and ALS. This paper presents the processing pipeline developed and intended for deployment in the BRAINTEASER project. This pipeline incorporates state-of-the-art techniques and addresses the requirements for analyzing these signals in a real-world consumer scenario. While numerous current techniques concentrate on high-frequency data obtained from specialized and case-specific sensors, this approach utilizes a readily accessible consumer device and their built-in sensors and features, such as optical sensors and pulse-oximeters. During the development and signal selection process, it was crucial to incorporate input from clinicians to ensure the extraction of necessary features and focus on the most relevant signals for the specific situation. The selection process also considered additional engineering and usability factors. However, as a result, the accelerometer data was discarded. It is worth noting that the readings from the accelerometer can be valuable for a variety of functionality assessments, particularly those related to motor skills. This interdisciplinary approach is of paramount importance to guarantee that the processing is performed on valuable signals for the specific case. This process results in the extraction of useful features that can be utilized in subsequent analysis steps or, for example, displayed in a monitoring interface.

The proposed pipeline has been developed to work on data covering one day, but it can also process data in shorter time windows. However, when using shorter time windows, the consistency of certain features should be ensured the consistency of certain features. Regardless of the time window, patterns over multiple days can be analyzed at a later stage by comparing the extracted features and metrics as necessary. The proposed pipeline will be implemented in the BRAINTEASER project and continuously refined to meet the specific requirements of its application in the project. Future work will involve examining the noise characteristics of data obtained from wearable devices and evaluating various advanced noise-filtering approaches. One possible approach is to utilize Kalman filtering, as investigated in the study cited in (35), to assess its potential to enhance the heart rate signal in this processing pipeline. Additionally, new features will be explored and developed to expand the capabilities of the pipeline beyond the current processing task. One specific area of improvement is the gap-filling technique, particularly in sleep-related signals. By using Bayesian approaches based on imputation of earlier periods, it may be possible to take into account patient habits and routines, which could enable the development of a priori information that is relevant for both imputation and long-term statistics. This could allow for better exploration of signal patterns, and more accurate analysis of the signals. Furthermore, the long-term statistics generated by the pipeline could provide a deeper understanding of the signals and could potentially aid in the detection of outliers, which may indicate significant health events. The developed pipeline is a useful tool for effectively utilizing consumer smartwatches in health monitoring, and it might enable improved monitoring and signal analysis of both sick and healthy individuals in various scenarios.

## Data Availability

The original contributions presented in the study are included in the article/Supplementary Material, further inquiries can be directed to the corresponding author/s.
